# Social Pharmacology as an Underappreciated Field in Medical Education: A Single Medical School’s Experience

**DOI:** 10.3389/fphar.2021.714707

**Published:** 2021-08-16

**Authors:** Jannis S. Papadopulos, Alexios-Fotios A. Mentis, Charis Liapi

**Affiliations:** ^1^ Medical School, University of Athens, Athens, Greece; ^2^ University Research Institute of Maternal and Child Health and Precision Medicine, National and Kapodistrian University of Athens, Athens, Greece; ^3^ Department of Pharmacology, Medical School, National and Kapodistrian University of Athens, Athens, Greece

**Keywords:** social pharmacology, social medicine, precision medicine, Greece, medical education

## Introduction

The social aspects of medicine and medical education are more than imperative nowadays, given that several societal determinants, such as health care systems, political settings, unemployment, racial inequities, and exposure to chronic stress (Chrousos, 2009; Theoharides, 2020), could play a major role in public attitudes towards therapies or even change the course of medical decisions (Russell et al., 2004; McNeill et al., 2017; Nuwayhid and Zurayk, 2019; Ghilardi et al., 2020). It is now equally appreciated that the drug discovery processes (Kaitin and DiMasi, 2011), which may disproportionally focus on some fields (e.g., oncology), should take into account societal factors (e.g., access to medications) (Milne and Kaitin, 2019). Previous studies have shown that the quality of higher education (including medical schools) appeared suboptimal in terms of addressing the current needs of patients and the broader society (Kulasegaram et al., 2013; Bakoyiannis et al., 2017). Therefore, the need to familiarize medical doctors and, in particular, medical students to these societal concepts and aspects is more crucial than ever.

Drugs are an undeniably chief tool for doctors in treating patients; however, the extent to which pharmacology courses address the ELSI (ethical, legal, and societal) aspects of pharmacology beyond providing strictly medical and scientific learning material (e.g., a drug’s mechanisms of action, its pharmacodynamics, pharmacokinetics, side effects) is largely explored, at least based on our experience at the National and Kapodistrian University of Athens Medical School. In general, a medical school curriculum consists of both basic and clinical medical science courses (e.g., biochemistry and surgery, respectively) which mostly focus on human physiology and diseases. We have also noticed, at least based on our experience, that in most courses, an underappreciated gap between pure biomedical and ELSI knowledge is present in medical education. This reflects the low exposure of students at medical schools, especially during their preclinical years of medical education, and students of pharmacy to societally- and humanities-focused courses, such as bioethics, health care, public health, health policy, medical history, or biomedical communications (Bell et al., 2008; Al-Haqwi and Al-Shehri, 2010; Westerhaus et al., 2015; Greenberg et al., 2016; Inerney, 2018). As a result, medical students and students of pharmacy may appear highly knowledgeable of drug-related mechanistic aspects but not significantly aware of societally-focused notions such as inappropriate prescription of drugs and/or the so-called *medicamentation*, which is defined as *the use of drugs for social problems previously not requiring drug utilisation* (*e.g*., *ageing, loss of libido, etc.*) (Mbongue et al., 2005). The above gap in education of students from schools of medicine and of pharmacy has been similarly observed in several other countries, implying it is a broader issue (Harding and Taylor, 2006; Hassali et al., 2011; Jaber et al., 2015; Makris et al., 2015; Mobrad et al., 2020; Góralska et al., 2021). Therefore, the lack of knowledge and skills in ELSI may, in turn, lead to suboptimal medical practice towards vulnerable groups. In fact, contemporary data on drug misuse (Culberson and Ziska, 2008), polypharmacy linked to multimorbidity (Blum et al., 2021), inappropriate and/or non-evidence-based self-medication (Yousef et al., 2008; Rather et al., 2017), misdosing (Nemati et al., 2016), inappropriate prescription trends (Hovstadius et al., 2014; Moriarty et al., 2015) (both hold true especially in the elderly), and mortality and morbidity outcomes in relation to drug overuse such as for opioids (Cheatle, 2015), alongside suboptimal medical practice (Ma et al., 2005; Massey et al., 2009), reinforce more than ever the call-to-action for developing Social Pharmacology courses to address these alarming issues.

*Social Pharmacology* aims to bridge the gap between biomedical knowledge/drug innovation and implementation of pharmacology to daily life. Implementers and/or educators must take into consideration how drugs are connected to society, notably 1) how social determinants, beyond medical or scientific factors, affect the use of drugs, 2) the social effects of drug use, 3) how the different stakeholders such as the pharmaceutical industry, regulatory agencies, sponsors, payers/public and even non-traditional stakeholders, such as lawyers and media, can direct decisions that impact society (as illustrated in the COVID-19 pandemic), and 4) how the patient’s or physician’s personal and social factors (e.g., cultural background, depth of medical knowledge, or social status) can affect drug use (Montastruc, 2002). To our best knowledge, with regards to Pharmacology courses, there is a major overlapping between medical schools and schools of pharmacy in their approach and curricula. In regards to societal aspects of the course of Pharmacology, although several issues (e.g., dietary management, exercise, digital therapeutics, etc.) are neglected in both schools, the major concern is the financial burden of medications but from different aspects, i.e., medical schools focus on patient and the health systems, while pharmacy schools tend to focus more on how the pharmacist will effectuate the doctor’s prescription and be compensated (Hassali et al., 2011; Sheridan, 2015). Moreover, we feel that public health courses’ curriculum is already loaded with a whole range of topics (e.g., epidemiology, biostatistics, bioethics, history of public health, global health, infectious diseases) and, in doing so, may not provide the necessary time and niche for including and discussing Social Pharmacology topic. Hence, we opted to focus on Social Pharmacology for medical students as future drug prescribers, hoping that our Commentary will make a case for more wide-spread inclusion of courses focusing exclusively on Social Pharmacology courses in medical schools. We took into account that, at least in Greece, there are no interdisciplinary programs in the medical school curriculum that enable the discussion of ELSI topics, social determinants of health, and other societal topics [e.g., racism (White-Davis et al., 2018)] in the delivery of medical care.

Within this context, several years ago at the Medical School of National and Kapodistrian University of Athens, Greece, we introduced and developed the course *Social Pharmacology*, an elective course for medical students during the preclinical years of medical education (i.e., during the equivalent of premedical (*premed*) courses in United States) (Liapi and Papadopulos, 2004). This course was based on previous, important theoretical and practical work in the field (Venulet, 1974; Venulet and Jucker, 1978; Alloza, 1987; Lilja and Larsson, 1994; Lilja et al., 1997; Morgan and Zimmer, 1997; Montastruc, 2002; Morgan, 2016). In particular, much of the course material was guided by previous, major publications in the field (Alloza, 2014; Maiti and Alloza, 2014; Gascón-MolinsJLAy, 2021). Notably, the so-called *European Higher Education Area*, which originated in the Bologna Declaration (June 19th, 1999) for European educational system reformation, includes Social Pharmacology as a subject of study within the Pharmacology program for medical students. However, at least in our experience and to our best knowledge, the current gap between medically-focused and ELSI subjects is still prevalent given that Social Pharmacology as a core course is still not included in many medical schools mostly due to downplaying of social pharmacology and the high pressure and demand by medical student for clinically-oriented academic courses.

The principal goal of this course was to familiarize budding medical doctors to the societal issues of drug prescription and use. In parallel, the goal of the course was also to familiarize the greater percentage of medical doctors and the society to the history and social aspects of pharmacology. To this end, we founded the *Museum of Pharmacology* (https://www.uoa.gr/to_panepistimio/moyseia/farmakologias/) (Papadopulos et al., 2004; Liapi, 2021a; Liapi, 2021b), the first-of-its-kind at least in Greece, to fulfill the pedagogic goals of the above course and to serve as an educational point of reference for Greece’s population of medical students and future physicians.

In the current Opinion article, we present our medical school’s efforts on Social Pharmacology; in particular, we describe our experience on establishing the course of Social Pharmacology, now spanning more than 2 decades, that aims to address the ESLI gap of Pharmacology in Greece, and we also reflect on the broader implications of Social Pharmacology in medicine.

## Social Pharmacology Course in Greece

The *Social Pharmacology* course, a one-semester elective long course established 2 decades ago and taught until some years ago in our Medical School, has aimed to link knowledge on pharmacology to *real life* (*pragmatic approach*) and not merely to treatment of diseases, has brought awareness to factors that are often neglected during medical decisions, and emphasized the need for strong relationships between doctors and patients.

The issues discussed in this elective course included: 1) misuse and overuse of drug prescriptions; 2) use of drugs in specific population groups and periods of life, i.e., elderly population, children, pregnancy, lactation; 3) effect of drug consumption on specific events (e.g., falls, car accidents); 4) drugs and alcohol use while driving; 5) alcohol and illicit drug use patterns and dependency in relation to social class, race, and gender; 6) doping and illicit drugs; 7) drug prescription and racial inequity; 8) drug prescription in people with disabilities (e.g., visually impaired); 9) compliance issues; 10) drug prescription as a substitute of doctor’s limited time; 11) the role of the pharmaceutical industry in providing objective and unbiased drug information; 12) unconscious bias from physicians during drug prescription; and, 13) prevention of accidental poisoning in Greece (it occurs at an incidence of ∼30,000 cases per year in the country).

To fulfill the course objectives, medical students were expected to be equipped with both the theoretical background and the hands-on skills concerning social pharmacology as applied in several settings, discuss and explore bioethical concerns related to pharmacology, in particular how drugs affect human behavior and society, foster intellectual curiosity, encourage questioning, and seek resolution of confusing information, analyze the impact of legal−ethical considerations on use of drugs, think openly and in an unbiased fashion about the intersection of prescription drug use and a patient’s societal milieu. Also, medical students were taught updated health-promoting techniques, prevention of illicit drugs use, as well as physical rehabilitation methods for people with disabilities.

It is now appreciated that teaching only through live academic lectures is not the most efficient way to stimulate students’ interest (Moreno López et al., 2009; Ronchetti, 2010). Therefore, several educational approaches were employed during the course of Social Pharmacology in order to stimulate medical students’ interest in that course: lectures, debates-discussions, video presentations, movies, field trips to various work areas (e.g., Alcoholic Anonymous (AA) or/and to Narcotic Anonymous), and last, group and individual projects. One major experience involved visit to a pharmaceutical company where students witnessed how drugs were manufactured by spending a whole day observing the production pipeline step-by-step; students ultimately appreciated the special precautions taken in order to avoid mistakes that could potentially results in a dangerous lot (e.g., active substance in lower quantity, wrong labeling, inappropriate excipient) (Papadopulos, 1995). In another field trip, students were familiarized with the real circumstances under which factory employees worked and became more aware for the importance of societal factors on prescribing and medical care. For instance, students appreciated that falls from a ladder under the influence of medications (e.g., use of anti-histamines in builders or lifting-machine operators) are not rare and that they must consider the working environment when prescribing medications. As another didactic experience, medical students were also taught to take care of people with disabilities. In this way, medical students learned how to better serve people with disabilities by taking into account specific factors while prescribing drugs such as existing morbidity (e.g., deafness, blindness, etc.); for instance, all prescriptions should be prepared in written for people with deafness and should be recorded for those with blindness and/or impaired vision. Finally, medical students were exposed to other, broader notions of public health, including, for example, the role of the built environment on people’s health (e.g., school buildings, playgrounds, etc.).

Other didactic examples of the student experience during the Social Pharmacology course are illustrated below. To better inform patients on how the drug can affect safety while driving, medical students were educated to identify specific adverse effects (e.g., hypotension, dizziness) when prescribing drugs to drivers, rather than simply reading bold indications for driver-safety issues. Students were also exposed to other potentially neglected topics, for instance how to advise patients to store drugs at a safe place at home and far from children’s attention to avoid accidental poisoning and/or suicide attempts. Another major area of focus in this course involved students’ learning skills in prescribing certain medicines (e.g., prescribing no more than a certain amount of paracetamol). Moreover, medical students were familiarized with several critical factors (such as the effect of age, level of education, urban *vs.* rural living) that can affect adherence to a pharmaceutical treatment (e.g., antihypertensive treatment as a case of non-compliance) (Yiannakopoulou et al., 2005).

Last, medical students were offered opportunities to harness their critical thinking skills in cases of real-world drug uses. Examples of these opportunities include 1) evaluating the drug leaflets to assess whether any ethics were violated while promoting a certain drug; 2) assessing the level of access of patients to information regarding available drug stores during the 24 h cycle; 3) familiarizing with quality standardization practices, malpractice linked to drug abuse and/or any concern on drug development regulatory issues; 4) appreciating the role of racial, ethnic, and religious factors while prescribing drugs (e.g., different racial groups may metabolize drugs differently due to distinct genetic ancestry background); and, 5) assessing how certain occupational categories may present different drug metabolism and enzyme induction profile (Uppal et al., 1982; Dossing, 1983). As a result, at the end of the course, students developed advanced problem-solving skills in patient-facing scenarios, in comparison to conventional teaching, and they also appreciated the wide scope and profound importance of regulatory and quality assurance issues in drug manufacturing and beyond.

To further develop the course’s goal and aims, the Museum of Pharmacology was founded to serve as a pedagogical tool for the course of Social Pharmacology (Papadopulos et al., 2004; Liapi, 2021a). In particular, the aims of the *Museum of Pharmacology,* which was founded 17 years ago inside the University of Athens Medical School’s campus, have been to 1) encourage academic research and active learning on Social Pharmacology-related topics; 2) understand and appreciate how laboratory equipment has progressed from traditional methods to more current, high-throughput technologies (e.g., a series of older and newer models of balances, centrifuges, etc.); 3) compare the scientific methods of experimental *vs.* social pharmacology, which focus on laboratory data vs*.* societal factors, respectively; 4) educate in the history of pharmacology from antiquity till now; and, last but not least, 5) serve as a virtual Museum. In the Museum, in close adherence to its mission, more than 600 historical and medically relevant items are exhibited, including research apparatus, medical pharmacology books, pharmacological research-related material, materials temporarily on loan from other museums. The Museum has also collaborated with similar institutions (e.g., other museums in medical schools) at both the national and international level towards the common goal of promoting Social Pharmacology. Moreover, the museum committee has organized seminars, lectures, guided tours, and additional academic activities aiming to foster the transfer of knowledge stemming from the Museum’s exhibits and highlight the importance of *Social Pharmacology*.

Two rather noteworthy examples referring to the Museum’s exhibits refer to 1) a poster presentation dating back to 1929, which advertises heroin as a solution to cough; heroin, before the addictive effects were known, was advertised as a better alternative to morphine for treating cough (Sneader, 1998; Art and Culture, 2008); and, 2) to posters for thermal springs as part of alternative medicine, as well as several posters aiming to discourage people from using illicit drugs.

Several reflections can be made after visiting the Museum: 1) appreciating how devices of the past that were once deemed useful (e.g., for drug manufacturing) are now obsolete and importantly, no longer used because they have been replaced by novel, more user-friendly and more automated devices, and importantly, no longer used; 2) comparing the lack of standardized and regulated clinical trials even 60 years ago (notably, before the thalidomide incident around the 1960s) vs*.* the high-cost, technology-driven trials of today, which may not be affordable for resources-poor institutions (Chrousos et al., 2020a); 3) delving into the drug manufacturing process (e.g., identifying problems that are often hidden behind a complete and apparently simple product); 4) understanding how medical needs are historically served by these drugs; and, 5) reflecting on the reproducibility of medical research (e.g., how *excellent results* can be proven false within just 10 years after their initial publication).

## Discussion

In the current Opinion article, we commented on our course on *Social* Pharmacology and the associated Museum of Pharmacology. This project can be used as a potential example for similar efforts in other countries. In particular, the course, upon completion, has been positively commended by medical students in their written evaluations. These students, based on the opinions of faculty members, were more motivated, resourceful, organized, and dedicated to practice medicine with high concern for the society’s needs. We feel that other medical schools, which have not already included ELSI-driven courses, should endorse at least some of the above components to their medical curriculum. We have received informal oral feedback on the clinical merit of this course, and the importance of the most recent approaches in health care and health care education, which aim to be patient-, human-, and society-centered (Carter et al., 2018; Kaitin, 2019).

Of note, some of the future challenges in medicine refer to how molecular and precision medicine should work hand-in-hand with social equity-based health care and how resource-poor countries can cope with biomedical advancement, in which the discoveries and implementation require extensive funding (Konstantinopoulos et al., 2009; Mentis et al., 2018; Chrousos et al., 2020b). Thus, our message of *Social Pharmacology* cannot be timelier. Importantly, courses and initiatives that are similar to those described above can serve as an appropriate starting point to address these complex issues and cultivate society-centered medical learning.

Last, but not least, additional new avenues of *Social Pharmacology* could include 1) how smart phone-applications, social media, and online social networks can affect and help trace the use and consumption of pharmaceutical products; 2) how lifestyle drugs that aim to enhance personal well-being (with potential sequelae on the societal level) can challenge the very notion of *Social Pharmacology* by creating a need to redefine drug indications (Flower, 2004); and, 3) how social conditions (e.g., housing conditions) can affect psychological behavior which, in turn, can have an impact on drug use (Bardo et al., 2013). These are all critical points which budding physicians-investigators and social pharmacologists could investigate.
